# Lipid-Based Drug Delivery Systems

**DOI:** 10.1155/2014/801820

**Published:** 2014-05-19

**Authors:** Hina Shrestha, Rajni Bala, Sandeep Arora

**Affiliations:** Chitkara College of Pharmacy, Chitkara University, Chandigarh-Patiala National Highway, Rajpura, Patiala, Punjab 140401, India

## Abstract

The principle objective of formulation of lipid-based drugs is to enhance their bioavailability. The use of lipids in drug delivery is no more a new trend now but is still the promising concept. Lipid-based drug delivery systems (LBDDS) are one of the emerging technologies designed to address challenges like the solubility and bioavailability of poorly water-soluble drugs. Lipid-based formulations can be tailored to meet a wide range of product requirements dictated by disease indication, route of administration, cost consideration, product stability, toxicity, and efficacy. These formulations are also a commercially viable strategy to formulate pharmaceuticals, for topical, oral, pulmonary, or parenteral delivery. In addition, lipid-based formulations have been shown to reduce the toxicity of various drugs by changing the biodistribution of the drug away from sensitive organs. However, the number of applications for lipid-based formulations has expanded as the nature and type of active drugs under investigation have become more varied. This paper mainly focuses on novel lipid-based formulations, namely, emulsions, vesicular systems, and lipid particulate systems and their subcategories as well as on their prominent applications in pharmaceutical drug delivery.

## 1. Introduction

In these modern days, many significant efforts have been applied to use the potentials of lipid-based drug delivery systems, as it provides the suitable means of site specific as well as time controlled delivery of drugs with different molecular weight, either small or large, and also the bioactive agents [1, 2]. Poorly water-soluble drugs are challenging for the formulation scientists with regard to solubility and bioavailability. Lipid-based drug delivery systems (LBDDS) have shown the effective size dependent properties so they have attracted a lot of attention. Also LBBDS have taken the lead because of obvious advantages of higher degree of biocompatibility and versatility. These systems are commercially viable to formulate pharmaceuticals for topical, oral, pulmonary, or parenteral delivery. Lipid formulations can be modified in various ways to meet a wide range of product requirements as per the disease condition, route of administration, and also cost product stability, toxicity, and efficacy. Lipid-based carriers are safe and efficient hence they have been proved to be attractive candidates for the formulation of pharmaceuticals, as well as vaccines, diagnostics, and nutraceuticals [3]. Hence, lipid-based drug delivery (LBDD) systems have gained much importance in the recent years due to their ability to improve the solubility and bioavailability of drugs with poor water solubility.

## 2. General Routes of LBDDS

Routes like oral, parenteral, ocular, intranasal, dermal/transdermal, and vaginal can be for the administration of the lipid-based drug delivery systems (LBDDS) [4, 5]. However, oral route is the most preferred route because of the properties like noninvasiveness, less expensive, and less prone to side effects, such as injection-site reactions. It is also considered as the easiest and the most convenient method of drug delivery for chronic therapies. But, at a very early stage of development, formulation strategies based on a rational and systematic approach need to be developed to avoid erratic and poor* in vitro/in vivo* correlations and thus increase the chances of success in formulation development. Various useful guidelines regarding the convenient routes and formulation strategies have been published by several authors [6–9].

## 3. Lipid Formulation Classification System

The lipid formulation classification system (LFC) was introduced as a working model in 2000 and an extra “type” of formulation was added in 2006 [10]. In recent years the LFCs have been discussed more widely within the pharmaceutical industry to seek a consensus which can be adopted as a framework for comparing the performance of lipid-based formulations. The main purpose of the LFCs is to enable* in vivo* studies to be interpreted more readily and subsequently to facilitate the identification of the most appropriate formulations for specific drugs, that is, with reference to their physiochemical properties as depicted in Table 1.

## 4. Points to Be Considered for the Formulation

Main factors affecting the choice of excipients for lipid-based formulations are as follows:

- solubility,
- dispersion,
- digestion,
- absorption.

Other factors are as follows:

- regulatory issues-irritancy, toxicity, knowledge, and experience,
- solvent capacity,
- miscibility,
- morphology at room temperature (i.e., melting point),
- self-dispersibility and role in promoting self-dispersion of the formulation,
- digestibility and fate of digested products,
- capsule compatibility,
- purity, chemical stability,
- cost of goods.

### 4.1. Solubility

While the lipids (fatty acid derivatives) are the core ingredient of the formulation, one or more surfactants, as well as perhaps a hydrophilic cosolvent, may be required to aid solubilization and to improve dispersion properties. Surfactants are categorized by their hydrophilic-lipophilic balance (HLB) number, with a low value (≤10) corresponding to greater lipophilicity and a higher value (≥10) corresponding to higher hydrophilicity. As a guideline as a starting point for formulation design, most of the lipids used in these oral formulations have a known “required HLB” value (generally available from the vendors), which corresponds to the optimal HLB for the surfactant blend necessary to emulsify the oil in water. Various emulsifiers can be used for the various formulations depending on their HLB values as depicted in Table 2 [11–13].

### 4.2. Dispersion

**Figure 1:**
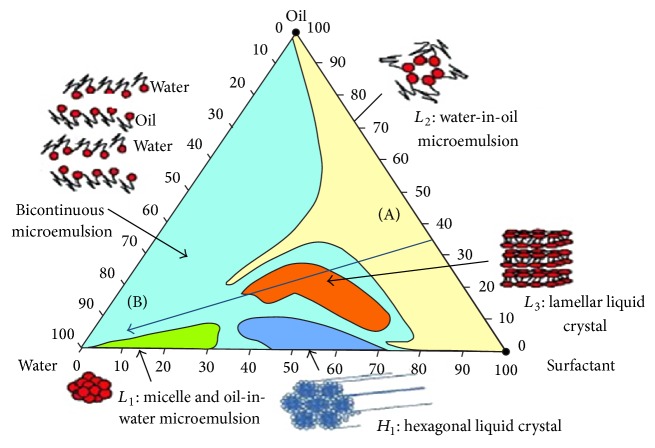


Formulations that exhibit sufficient solubility of the drug candidate should be examined for emulsification and dispersion properties in aqueous vehicles. A preliminary screening can be carried out by microscopic observation of the formulation when mixed with water. Vigorous mixing, accompanied by diffusion and stranding mechanisms, occurring at the water/formulation interface is indicative of an efficient emulsification. Absence of drug precipitate after complete mixing of the formulation with aqueous medium is another requirement. Particle size measurement of emulsion droplets by laser light scattering or other techniques is useful to select promising formulations. Construction of ternary phase diagrams is a method frequently used to determine the types of structures resulting from emulsification and to characterize behavior of a formulation along a dilution path. An example is shown in Figure 1; the line from A to B represents dilution of a formulation consisting initially of 35% surfactant, 65% oil, passing through regions of a water-in-oil microemulsion and a lamellar liquid crystal until reaching a stable bicontinuous oil-in-water microemulsion after dilution. It is often unnecessary to construct the entire phase diagram, but an understanding of the structures arising on a dilution path of a given formulation is important to assure formation of stable dispersed structures upon dilution. Appropriate combinations of low HLB and high HLB surfactants frequently lead to smaller emulsion droplet size than single surfactants. These more complex combinations can be examined by pseudoternary phase diagrams [13].

### 4.3. Digestion

The actions of intestinal lipases can have a profound effect on the behavior of lipid-based formulations in the GI tract and must be considered in their design. It has long been recognized that nondispersible but digestible lipids such as triglycerides can be metabolized by lipases to mono-/diglycerides and fatty acids which will emulsify any remaining oil. Thus, the presence of high amounts of surfactants may be unnecessary to assure creation of the requisite small particle sizes and large surface areas for drug release. In 2000, Pouton proposed a classification system for lipid-based formulations based on the formulation components and the dependence on digestion to facilitate dispersion [14], which is shown in Table 3.

### 4.4. Absorption

**Figure 2:**
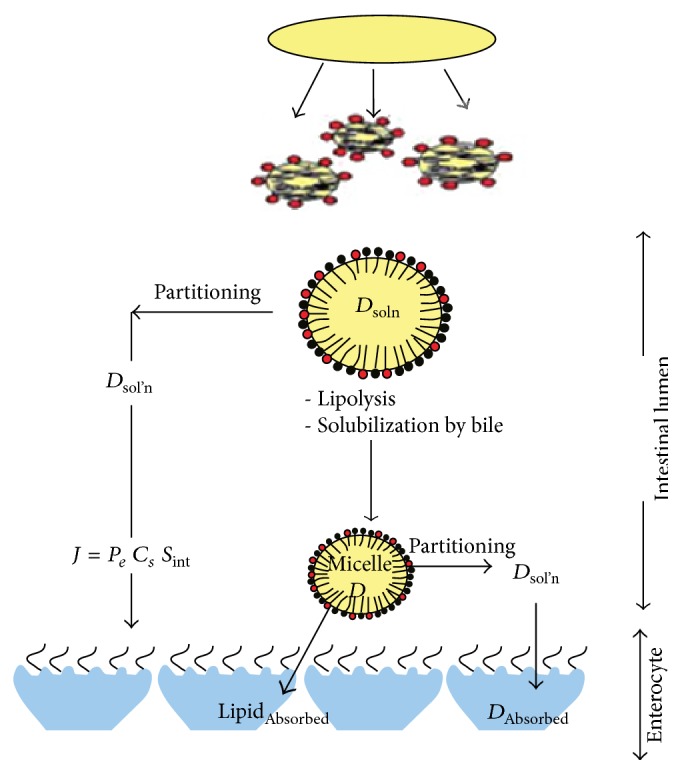


Efficient absorption of the drug by the intestinal mucosal cells is of course the ultimate goal of any oral lipid-based formulation.  Figure 2 shows the processes that occur in the intestinal milieu for a lipid-based drug formulation [13]. First the components are dispersed to form lipid droplets (for type I formulations) or emulsion droplets (for types II-III), followed by lipolysis and solubilization of the digestion products by bile acids, forming colloidal mixed micelles. It is believed that drug then partitions from the emulsion oil droplets and bile salt mixed micelles to be absorbed by the mucosal cells of the intestinal wall.

## 5. Advantages of LBDDS [15]

1. Drug release in controlled and targeted way.
2. Pharmaceutical stability.
3. High and enhanced drug content (compared to other carriers).
4. Feasibilities of carrying both lipophilic and hydrophilic drugs.
5. Biodegradable and biocompatible.
6. Excipients versatility.
7. Formulation versatility.
8. Low risk profile.
9. Passive, noninvasive formation of vesicular system which is available for immediate commercialization.

## 6. Types of Lipid-Based Drug Delivery Systems

**Figure 3:**
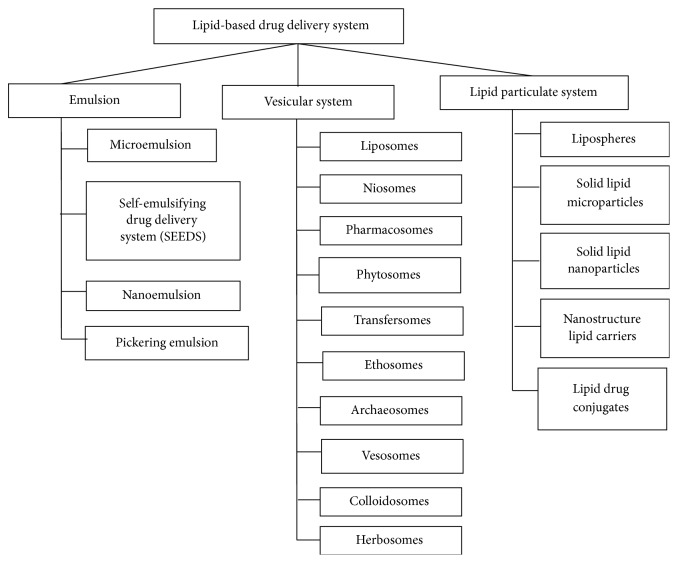


For more details see Figure 3.

## 7. Guidelines for Design of Lipid-Based Formulations

While it is apparent that lipid-based formulations will continue to be an important tool to formulate poorly soluble drugs, design of these formulations can be a challenge. In their excellent review, Porter et al. [16] recently outlined seven guidelines for design of lipid-based formulations, as summarized below.

1. It is critical to maintain drug solubility in the formulation, after dispersion, and after digestion.
2. Properties of the colloidal species formed after processing in the GI milieu are probably more important than properties of the formulation itself in enhancing absorption.
3. Higher proportions of lipid (>60%) and lower proportions of surfactant (<30%) and cosolvent (<10%) generally lead to more robust drug solubilization after dilution.
4. Medium chain triglycerides may afford greater drug solubility and stability in the formulation, but long chain triglycerides facilitate more efficient formation of bile salt lipid colloidal species and thus may afford higher bioavailability.
5. Type IIIB SMEDDS formulations give lower droplet sizes after dispersion. However, they are more dependent on the surfactant properties employed, and nondigestible surfactants generally give higher bioavailability.
6. Dispersion of type IV formulations (surfactant/cosolvent) is likely more efficient if two surfactants are used rather than a single one.
7. Type IV formulations may give higher drug solubility but must be designed carefully to assure that drug does not precipitate after dispersion.

These guidelines are important ones to keep in mind when designing oral lipid-based formulations for poorly soluble drugs. As further experience is gained with design and use of these formulations and the database of successful formulations grows, it is to be hoped that design of these formulations will become less of an empirical exercise and more rational in its approach. As this happens, the utility of lipid-based formulations can only grow.

## 8. Formulation Approaches for LBDDS

### 8.1. Spray Congealing

This is also referred to as spray cooling. In this method, molten lipid is sprayed into a cooling chamber and, on contact with the cool air, congeals into spherical solid particles. The solid particles are collected from the bottom of the chamber, which can be filled into hard gelatin capsules or compressed into tablets. Ultrasonic atomizers are frequently used to produce solid particles in this spray cooling process. The parameters to be considered are the melting point of the excipient, the viscosity of the formulation, and the cooling air temperature inside the chamber to allow instant solidification of the droplets.

### 8.2. Spray Drying

This method is somewhat similar to preceding one but differs in the temperature of the air inside the atomizing chamber. In this method, the drug solution (drug in organic solution/water) is sprayed into a hot air chamber, where the organic solvent or water evaporates giving rise to solid microparticles of drug. During this process, along with the lipid excipients, solid carriers like silicon dioxide can be used. Gelucire (lipid excipient) enhances the drug release process by forming hydrogen bonds with the active substance, leading to the formation of stable solids of amorphous drug in microparticles [17, 18].

### 8.3. Adsorption onto Solid Carrier

This is a simple and economical process (in the context of equipment investment) in which a liquid-lipid formulation is adsorbed onto solid carrier like silicon dioxide, calcium silicate, or magnesium aluminometasilicate. The liquid-lipid formulation is added to the carrier by mixing in a blender. The carrier must be selected such that it must have greater ability to adsorb the liquid formulation and must have good flow property after adsorption. Gentamicin and erythropoietin with caprylocaproyl polyoxylglycerides (Labrasols) formulations were successfully converted into solid intermediates whose bioavailability was maintained even after adsorption on carriers. Advantages of this method include good content uniformity and high lipid exposure [19–21]. Ito et al. have developed a solid formulation of gentamicin using emulsifier and adsorbent. Using solid adsorbents like calcium silicate, magnesium aluminometasilicate, and silicon dioxide, the liquid mixture (drug and emulsifier like Labrasols) was converted to solid by a kneading process [21].

### 8.4. Melt Granulation

This is also referred to as pelletization, which transforms a powder mix (with drug) into granules or pellets [22–24]. In this method a melt able binder (molten state) is sprayed onto the powder mix in presence of high-shear mixing. This process can be referred to as a “pump on” technique. Alternatively, the melt able binder is blended with powder mix and, due to the friction of particles (solid/semisolid) during the high-shear mixing, the binder melts. The melted binder forms liquid bridges between powder particles and forms small granules which transform into spheronized pellets under controlled conditions. Depending on the fineness of the powder, 15%–25% of the lipid-based binder can be used. The parameters to be considered during the process are binder particle size, mixing time, impeller speed, and viscosity of the binder on melting [25]. The dissolution rate of diazepam was enhanced by formulating melt agglomerates containing solid dispersions of diazepam [26]. Lactose monohydrate was melt-agglomerated with a melt able binder like PEG 3000 of Gelucires 50/13 in a high-shear mixer. Polyoxylglycerides, partial glycerides or polysorbates, and lecithin are some of the lipid excipients used in the melt granulation technique to form self-microemulsifying systems [26, 27].

### 8.5. Supercritical Fluid-Based Method

This method uses lipids for coating drug particles to produce solid dispersions. In this method, the drug and lipid-based excipients are dissolved in an organic solvent and supercritical fluid (carbon dioxide) by elevating the temperature and pressure [28, 29]. The coating process is facilitated by a gradual reduction in pressure and temperature in order to reduce the solubility of the coating material in the fluid and hence precipitate onto the drug particles to form a coating [30, 31]. The solubility of the formulation components in the supercritical fluid and stability of the substance during the process are important considerations of this method.

### 8.6. Other Formulation Tools

Analysis of drug solubilization in bile salt-lecithin mixed micelles is an uncomplicated and effectual diagnostic test. Drug solubilization can be analyzed directly by spectrophotometry in some cases or alternatively by HPLC. This technique offers a rapid indication of whether a drug is likely to be solubilised in the gut lumen. The solubility enhancement ratio of steroids is a good illustration that solubilization cannot be predicted simply by octanol-water partition coefficient. Molecular dynamics modeling may become a useful formulation tool as available computing power increases. The structure of lipid formulations could be examined using similar techniques and studies of the partitioning [32].

## 9. Characterization of Lipid-Based Drug Delivery Systems

### 9.1. Appearance

The appearance can be checked in graduated glass cylinder or transparent glass container for its uniformity and colour at equilibrium [33].

### 9.2. Color, Odor, and Taste

These characteristics are especially important in orally administered formulation. Variations in taste, especially of active constituents, can often be accredited to changes in particle size, crystal habit, and subsequent particle dissolution. Changes in color, odor, and taste can also indicate chemical instability [34].

### 9.3. Density

Specific gravity or density of the formulation is an essential parameter. A decrease in density often indicates the entrapment air within the structure of the formulation. Density measurements at a given temperature can be made using high precision hydrometers [34].

### 9.4. pH Value

The pH value of aqueous formulation should be taken at a given temperature using pH meter and only after settling equilibrium has been reached, to minimize “pH drift” and electrode surface coating with suspended particles. Electrolyte should not be added to the external phase of the formulation to stabilize the pH, because neutral electrolytes disturb the physical stability of the suspension [34].

### 9.5. Self-Dispersion and Sizing of Dispersions

Assessment of the dispersion rate and resultant particle size of lipid-based systems is desirable so attention has been given to measuring dispersion rate. The particle size measurement can be performed by optical microscope using a compound microscope for the particles with measurement within microns. Particle size analyzer can be used for the measurement of the particle size.

### 9.6. Droplet Size and Surface Charge (Zeta Potential)

The droplet size distribution of microemulsion vesicles can be determined by either electron microscopy or light-scattering technique. The dynamic light-scattering measurements are taken at 90° in a dynamic light-scattering spectrophotometer which uses a neon laser of wavelength 632 nm. The data processing is done in the built-in computer with the instrument. Recently, with respect to the importance of particle size distribution in terms of particle characterization and product physical stability testing, there has been interest in newer light-scattering methods for particle detection called photon correlation spectroscopy (PCS).

The surface charge is determined using a zeta potential analyzer by measuring the zeta potential (ZP) of the preparations. ZP characterizes the surface charge of particles and thus it gives information about repulsive forces between particles and droplets. To obtain stable nanoemulsions by preventing flocculation and coalescence of the Nano droplets, ZP should typically reach a value above 30 mV [34].

### 9.7. Viscosity Measurement

Brookfield type rotary viscometer can be used to measure the viscosity of lipid-based formulations of several compositions at different shear rates at different temperatures. The samples for the measurement are to be immersed in it before testing and the sample temperature must be maintained at 37 ± 0.2°C by a thermo bath. The viscometer should be properly calibrated to measure the apparent viscosity of the suspension at equilibrium at a given temperature to establish suspension reproducibility. Apparent viscosity, like pH, is an exponential term, and therefore the log-apparent viscosity is a suitable way of reporting the results [34].

### 9.8. *In Vitro* Studies

*In vitro* evaluation of lipid-based drug delivery systems can be done with the use of lipid digestion models. In order to assess the performance of an excipient during formulation development and to predict* in vivo* performance, it is necessary to design an* in vitro* dissolution testing method. This can be termed as “simulated lipolysis release testing” [35]. The basic principle on which this system works requires maintaining a constant pH during a reaction which releases or consumes hydrogen ions. If any deviation is found, it is compensated by the reagent addition. The model consists of a temperature-controlled vessel (37 ± 1°C), which contains a model intestinal fluid, composed of digestion buffer, bile salt (BS), and phospholipid (PL). Into this model a fluid lipid-based formulation is added and to initiate the digestion process pancreatic lipase and colipase were added. As the digestion process starts it results in the liberation of fatty acids, causing a transient drop in pH. This drop in pH is quantified by a pH electrode. The pH electrode is coupled with a pH-stat meter controller and auto burette. An equimolar quantity of sodium hydroxide is added to titrate the liberated fatty acids by the auto burette, so as to prevent a change in pH of the digestion medium from a preset pH value. By quantifying the rate of sodium hydroxide addition and considering the stoichiometric relationship between fatty acids and sodium hydroxide, the extent of digestion can be quantized. During the digestion process, samples can be withdrawn and separated into a poorly dispersed oil phase, highly dispersed aqueous phase, and precipitated pellet phase by centrifugation. Quantification of drug in the highly dispersed aqueous phase indicates that drug has not precipitated, from which an assumption can be made with respect to* in vivo* performance of the lipid-based formulation.

### 9.9. *In Vivo* Studies

The impact of excipients on the bioavailability and pharmacokinetic profile of drugs can be estimated by designing appropriate* in vivo* studies. A detailed study of intestinal lymphatic absorption is required, since lipid-based formulations enhance bioavailability by improving the intestinal uptake of drug. Due to insufficient clinical data and differences in methods and animal models used, studies related to the drug transport by lymphatic system have become difficult [36].

### 9.10. *In Vitro*-*In Vivo* Correlation (IVIVC)

*In vitro*-*in vivo* correlation will help to maximize the development potential and commercialization of lipid-based formulations. A shortened drug development period and improved product quality could be achieved by developing a model that correlates the* in vitro* and* in vivo* data. Determining the solubility, dissolution, lipolysis of the lipid excipient, and intestinal membrane techniques (isolated animal tissue and cell culture models) are various* in vitro* techniques that can be used to assess lipid-based formulations [37]. Such techniques provide information about specific aspects of the formulation only. But it is important to know the* in vivo* interaction and performance of these systems. Similar to that of* in vivo* enterocytes, Caco-2 cells produce and secrete chylomicrons on exposure to lipids. More study has to be carried out on the choice of the most suitable* in vivo* model for assessing the lipid-based formulations.

## 10. Applications

- So far, the design of successful lipid-based delivery systems has been based largely upon empirical experiences. Systematic physicochemical investigations of structure and stability do not only help to speed up the development of new and improved formulations, but may also aid in the understanding of the complex mechanisms governing the interaction between the lipid carriers and the living cells. Hence they sought to be safe, efficient, and specific carriers for gene and drug delivery.
- LBDDS can be used to deliver various types of drugs from new chemical entities to more recent new developments for proteins and peptides, nucleic acids (DNA, siRNA), and cellular site specific delivery [38–40].
- The utility of lipid-based formulations to enhance the absorption of poorly water-soluble, lipophilic drugs has been recognized for many years. Lipids are perhaps one of the most versatile excipients classes currently available, providing the formulator with many potential options for improving and controlling the absorption of poorly water-soluble drugs. These formulation options include lipid suspensions, solutions, emulsions, microemulsions, mixed micelles, SEDDS, SMEDDS, thixotropic vehicles, thermosoftening matrices, and liposomes.
- Lipid-based formulations, which are by no means a recent technological innovation, have not only proven their utility for mitigating the poor and variable gastrointestinal absorption of poorly soluble, lipophilic drugs, but also, in many cases, have shown the ability to reduce or eliminate the influence of food on the absorption of these drugs. Despite these realities, marketed oral drug products employing lipid-based formulations are currently outnumbered 25 to 1 by conventional formulations. Some of the commercially available lipid-based formulations are depicted in Table 4.

## 11. Stability

Maintaining adequate chemical and physical stability of lipid-based drug formulations delivery systems can also present challenges like unsaturated lipid components which can undergo lipid per oxidation [41]. This can be minimized by use of saturated medium chain (C6-C12) triglycerides and by use of appropriate antioxidants. Phenol-based antioxidants such as Vitamin E (*α*-tocopherol), butylated hydroxytoluene (BHT), butylated hydroxyanisole (BHA), and propyl gallate can act synergistically with oxygen scavengers such as ascorbic acid and its lipid-soluble counterpart, ascorbyl palmitate.

## 12. Regulatory Aspects

Not all excipients are inert substances, and some may be toxic at augmented concentrations [42]. In the Code of Federal Regulations, the FDA has published a list of substances that are generally recognized as safe (GRAS). Apart from this, it also maintains a list of inactive ingredients for excipients entitled inactive ingredient guide (IIG) that are approved and can be incorporated in marketed products [43]. This guide provides the list of maximum amount allowed for excipients, which can be used for a specific route of administration. Once an inactive ingredient has been approved for a product through a particular route of administration, it can be used in any new drug formulation and does not require extensive review. The formulator can take the information from both GRAS and IIG when developing a new formulation. Currently, the FDA does not have any process or mechanism to evaluate the safety of excipients individually. Instead, the excipients are reviewed and approved as “components” of the drug or biological product in the application. Since excipients play an integral part in the formulation and cannot be reviewed separately from the drug formulation, the regulatory process is appropriate from a scientific standpoint.

From a regulatory point of view, quality and safety issues associated with preclinical and clinical studies are the key difficulties likely to be encountered in launching a lipid-based dosage form on the market, and above all the manifestations of the therapeutic efficacy. The overall drug stability and absence of immunological reactions to the oils or lipid excipients have to be demonstrated. Sufficient details explaining the use of lipid excipients and the types of dosage form, the drug release mechanism, and their manufacture should be provided to persuade the regulatory authorities of their acceptability [44]. Safety assessment and the potential influence of biopharmaceutical factors on the drug or lipid excipients need to be explored. It may be difficult to predict* in vivo* performances of a lipid dosage form based on* in vitro* results obtained through conventional dissolution methods in view of the convoluted GI processing of lipid formulations. More mechanistic studies should be conducted to facilitate a better understanding of the pharmaceutical characteristics of lipid formulations and interactions between lipid excipients, drug, and physiological environment. The lack of predictability for product quality and performance may be due to the nature of empirical and iterative processes traditionally employed [45].

With the aim of rationalizing the design of lipid formulation and to better understand the fate of a drug after oral administration in a lipid-based formulation, a consortium, composed of academics and industrial scientists, has been created (http://www.lfcsconsortium.org/). The consortium sponsors and conducts research to develop* in vitro* methods to assess the performance of LBDDS during dispersion and digestion, which are critical parameters. The primary objective is to develop guidelines that rationalize and accelerate the development of drug candidates through the identification of key performance criteria and the validation and eventual publication of universal standard tests and operating procedures. In order to establish approved guidelines, appropriate dialogue with pharmaceutical regulatory bodies (FDA, EMEA) is also foreseen.

## 13. Future Prospects

More consideration needs to be paid to the characteristics of various lipid formulations available, so that guidelines and experimental methods can be established that allow identification of candidate formulations at an early stage. Methods need to be sought for tracking the solubilization state of the drug* in vivo*, and there is a need for* in vitro* methods for predicting the dynamic changes, which are expected to take place in the gut. Attention to the physical and chemical stability of drugs within lipid systems and the interactions of lipid systems with the components of capsule shells will also be required. Whilst these present challenges there is a great potential in the use of lipid formulations. The priority for future research should be to conduct human bioavailability studies and to conduct more basic studies on the mechanisms of action of this fascinating and diverse group of formulations.

## 14. Conclusion

Lipid-based drug delivery systems provide the vast array of possibilities to formulations as they potentially increase the bioavailability of number of poorly soluble drugs along with the formulations of physiologically well tolerated class. The development of these systems requires proper understanding of the physicochemical nature of the compound as well as the lipid excipients and gastrointestinal digestion. One of the major challenges of lipid excipients and delivery systems is the varying range of compounds they contain. Proper characterization and evaluation of these delivery systems, their stability, classification, and regulatory issues consequently affect the number of these formulations. On the way of conclusion, the prospect of these delivery systems looks promising.

## Figures and Tables

**Table 1:** The lipid formulation classification system: characteristic features, advantages, and disadvantages of the four essential types of “lipid” formulations.

Formulation type	Material	Characteristics	Advantages	Disadvantages
Type I	Oils without surfactants (e.g., tri-, di-, and monoglycerides)	Nondispersing requires digestion	Generally recognized as safe (GRAS) status; simple; and excellent capsule compatibility	Formulation has poor solvent capacity unless drug is highly lipophilic

Type II	Oils and water insoluble surfactants	SEDDS formed without water-soluble components	Unlikely to lose solvent capacity on dispersion	Turbid o/w dispersion (particle size 0.25–2 *μ*m)

Type III	Oils, surfactants, and cosolvents (both water-insoluble and water-soluble excipients)	SEDDS/SMEDDS formed with water-soluble components	Clear or almost clear dispersion, drug absorption without digestion	Possible loss of solvent capacity on dispersion, less easily digested

Type IV	Water-soluble surfactants and cosolvents	Formulation disperses typically to form a micellar solution	Formulation has good solvent capacity for many drugs	Likely loss of solvent capacity on dispersion may not be digestible

**Table 2:** Emulsifiers used in lipid-based formulations.

Common name/type	Examples
Low HLB (<10) emulsifier	
Phosphatidylcholine and phosphatidylcholine/solvent mixtures	Phosphatidylcholine, phosphatidylcholine in propylene glycol, phosphatidylcholine in medium chain triglycerides, and phosphatidylcholine in safflower oil/ethanol
Unsaturated polyglycolized glycerides	Oleoyl macrogolglycerides, linoleoyl macrogolglycerides
Sorbitan esters	Sorbitan monooleate, sorbitan monostearate, sorbitan monolaurate, and sorbitan monopalmitate

High HLB (>10) emulsifier	
Polyoxyethylene sorbitan esters	Polysorbate 20, polysorbate 40, polysorbate 60, and polysorbate 80
Polyoxyl castor oil derivatives	Polyoxyl 35 castor oil, polyoxyl 40 hydrogenated castor oil
Polyoxyethylene polyoxypropylene block copolymer	Poloxamer 188, poloxamer 407
Saturated polyglycolized glycerides	Lauroyl macrogolglycerides, stearoyl macrogolglycerides
PEG-8 caprylic/capric glycerides	Caprylocaproyl macrogolglycerides
Vitamin E derivative	Tocopherol PEG succinate

**Table 3:** Pouton's classification of lipid-based delivery systems.

	Type I	Type II	Type IIIA	Type IIIB
% triglycerides or mixed glycerides	100	40–80	40–80	<20
% surfactants	—	20–60 (HLB < 12)	20–40 (HLB > 11)	20–50 (HLB > 11)
% hydrophilic cosolvents	—	—	0–40	20–50
Particle size of dispersion (nm)	Coarse	100–250	100–250	50–100
Significance of aqueous dilution	Limited importance	Solvent capacity unaffected	Some loss of solvent capacity	Significant phase changes and potential loss of solvent capacity
Significance of digestibility	Crucial requirement	Not crucial, but likely to occur	Not crucial, but may be inhibited	Not required and not likely to occur

**Table 4:** Some of the commercially available lipid-based formulations.

Molecules/trade name	Indication	Dose	Type of formulation	Lipid excipients and surfactants
Calcitriol/Rocaltrol	Calcium regulator	Adult: 0.25–0.5 *μ*g q.d.	Soft gelatin capsule	Fractionated triglyceride of coconut oil
Cyclosporin/Nerol	Immunosuppressant	2–10 mg/kg/day b.i.d.	Soft gelatin capsule	Cremophor RH 40
Tretinoin/Vesanoid	Antineoplastic	45 mg/m^2^ subdivided	Soft gelatin capsule	Bees wax, hydrogenated soybean oil
Valporic acid/Depakene	Antiepileptic	10–60 mg/kg/day	Soft gelatin capsule	Corn oil
Fenofibrate/Fenogal	Antihyperlipoproteinemic	200 mg q.d	Hard gelatin capsule	Gelucire 44/4
Testosterone/Restandol	Hormone replacement therapy	40–160 mg q.d.	Soft gelatin capsule	Oleic acid
